# Skull Fractures Induce Neuroinflammation and Worsen Outcomes after Closed Head Injury in Mice

**DOI:** 10.1089/neu.2019.6524

**Published:** 2019-12-20

**Authors:** Liga Zvejniece, Gundega Stelfa, Edijs Vavers, Einars Kupats, Janis Kuka, Baiba Svalbe, Baiba Zvejniece, Christiane Albert-Weissenberger, Anna-Leena Sirén, Nikolaus Plesnila, Maija Dambrova

**Affiliations:** ^1^ Latvian Institute of Organic Synthesis, Riga, Latvia.; ^2^ Latvia University of Life Sciences and Technologies, Jelgava, Latvia.; ^3^ Riga Stradins University, Riga, Latvia.; ^4^ University of Latvia, Riga, Latvia.; ^5^ Department of Neurosurgery, University Hospital Würzburg, Würzburg, Germany.; ^6^ University of Munich, Institute for Stroke and Dementia Research, Munich, Germany.

**Keywords:** neuroinflammation, skull fracture, traumatic brain injury, weight-drop model

## Abstract

The weight-drop model is used widely to replicate closed-head injuries in mice; however, the histopathological and functional outcomes may vary significantly between laboratories. Because skull fractures are reported to occur in this model, we aimed to evaluate whether these breaks may influence the variability of the weight-drop (WD) model. Male Swiss Webster mice underwent WD injury with either a 2 or 5 mm cone tip, and behavior was assessed at 2 h and 24 h thereafter using the neurological severity score. The expression of interleukin (IL)-6, IL-1β, tumor necrosis factor-α, matrix metalloproteinase-9, and tissue inhibitor of metalloproteinase-1 genes was measured at 12 h and 1, 3, and 14 days after injury. Before the injury, micro-computed tomography (micro-CT) was performed to quantify skull thickness at the impact site. With a conventional tip diameter of 2 mm, 33% of mice showed fractures of the parietal bone; the 5 mm tip produced only 10% fractures. Compared with mice without fractures, mice with fractures had a severity-dependent worse functional outcome and a more pronounced upregulation of inflammatory genes in the brain. Older mice were associated with thicker parietal bones and were less prone to skull fractures. In addition, mice that underwent traumatic brain injury (TBI) with skull fracture had macroscopic brain damage because of skull depression. Skull fractures explain a considerable proportion of the variability observed in the WD model in mice—i.e., mice with skull fractures have a much stronger inflammatory response than do mice without fractures. Using older mice with thicker skull bones and an impact cone with a larger diameter reduces the rate of skull fractures and the variability in this very useful closed-head TBI model.

## Introduction

Traumatic brain injury (TBI) may result from falls, motor vehicle accidents, sports injuries, and explosions and is one of the leading causes of neurological deficits in persons under the age of 45. The TBI is heterogeneous with many etiologies and clinical presentations and encompasses diffuse, focal, penetrating, or blast injury.^1,2^ Diffuse axonal injury results from movement of the brain within the skull and is related to closed-head injury (CHI), the most common type of TBI in humans.^3^ During the last decade, substantial attention has been paid to the study of TBI. Reproducible animal models are crucial to clarify the biochemical/molecular mechanisms of injury and to assess preclinical drug efficacy and safety.

Numerous animal models have been used to study TBI, including open and CHI.^4^ While mechanical force is delivered to the intact skull in CHI,^4^ in open head injury, a craniotomy has to be performed, and the impact is directed toward the dura mater.^5,6^ To perform CHI, several models are used, such as variations in weight-drop (WD),^5–7^ piston-driven,^8–10^ and blast injury^11^ TBI models. The WD models that use a free falling weight are used most widely to induce CHI.^12^

Although WD models have been used for several decades, there is a substantial difference in performance and protocols between laboratories. For example, the weight of the falling cylinder varies from 5 g to 500 g, and the drop height varies from 1 cm to 167 cm.^13–16^ As a consequence, the biochemical outcome— i.e., the expression of inflammatory cytokines, such as tumor necrosis factor (TNF)-α, interleukin (IL)-6, IL-12, and IL-1*β*, is highly variable.^17–21^

Controlled cortical impact and fluid percussion injury models, procedures that need a craniotomy, induce comparable high cytokine expression.^12,22–24^ Because in a previous study we observed that approximately 30% of mice experienced skull fractures after WD injury (unpublished data), we hypothesize that injury to the skull may be one of the main triggers for the expression of inflammatory genes in the brain after WD injury and may thus contribute to the variability observed in this valuable TBI model. Therefore, the aim of the present study was to compare the inflammatory response after WD injury in the hippocampus and striatum of mice and correlate these findings with the presence and severity of skull fractures.

## Methods

### Animals

One hundred and nine male Swiss Webster (Laboratory Animal Centre, University of Tartu, Tartu, Estonia) 10-week-old mice were used for the WD model and 13 male Swiss Webster 10- and 20-week-old mice were used for micro-computed tomography (micro-CT). The mice weighed 28–46 g; all animals were housed under standard conditions (21–23°C, 12 h light/dark cycle) with unlimited access to standard food (Lactamin AB, Mjölby, Sweden) and water.

The mice were assigned randomly to one of three experimental groups. Body weight was recorded throughout the study as a measure of general health. The experimental design was to expose 10-week-old mice to CHI using a weight-drop device with 2 or 5 mm cone tip or sham treatment. The skull was then examined for evidence of visible fractures, as defined below. Animals with CHI were subdivided into non-fracture and fracture (mild, moderate, severe) groups for comparison. The neurobehavioral status of the mice was obtained at 2 and 24 h after injury. Blood and brain tissue were collected at 12 h or 1, 3, and 14 days post-TBI for quantitative reverse transcription-polymerase chain reaction (RT-PCR) and enzyme-linked immunosorbent assay (ELISA), as described below. Before injury, a micro-CT was performed to quantify skull thickness at the impact site.

**FIG. 1.**
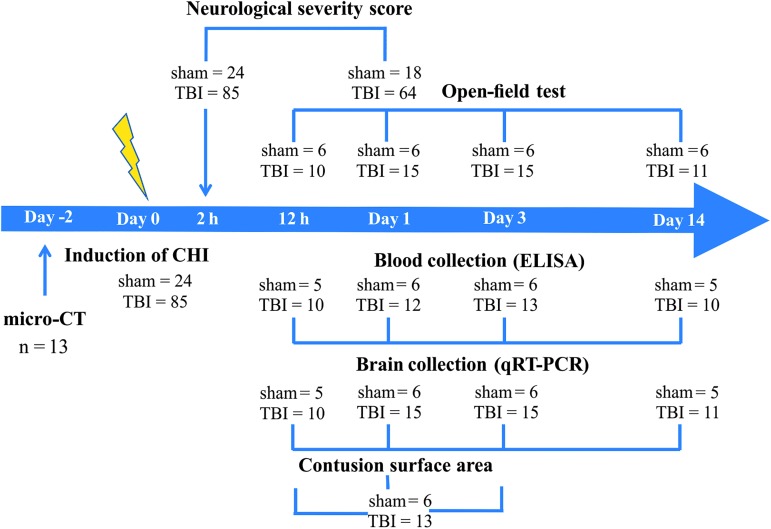
Schematic illustration of the experimental design. TBI, traumatic brain injury; CHI, closed head injury; ELISA, enzyme-linked immunosorbent assay; micro-CT, micro-computed tomography; qRT-PCR, quantitative reverse transcription: polymerase chain reaction. Color image is available online.

The timeline of experimental procedures is depicted in Figure 1. All studies involving animals were reported in accordance with the Animal Research: Reporting *In Vivo* Experiments guidelines.^25,26^ The experimental procedures were performed in accordance with the guidelines reported in the EU Directive 2010/63/EU and in accordance with local laws and policies; all procedures were approved by the Latvian Animal Protection Ethical Committee of Food and Veterinary Service in Riga, Latvia.

### Chemicals

Isoflurane was purchased from Chemical Point (Deisenhofen, Germany). Tramadol solution was purchased from KRKA (Novo Mesto, Slovenia). Physiological saline (0.9%) was purchased from Fresenius Kabi (Warsaw, Poland). Oxygen and nitrous oxide gases were purchased from AGA (Riga, Latvia).

### WD model

The WD model was performed as described previously.^5,6^ Briefly, the mice were anesthetized with 4% isoflurane in a mixture of 50% nitrous oxide and 50% oxygen, and 2% isoflurane was maintained during the surgical procedure using a face mask. The depth of anesthesia was monitored by toe pinch. Before trauma induction, the mice received a subcutaneous (sc) injection of tramadol (10 mg/kg). A midline longitudinal scalp incision was made, and the skull was exposed. A cone with a tip diameter of 2 or 5 mm was placed 2 mm posterior and lateral to bregma, and a weight of 90 g was dropped from a height of 8 cm onto the cone.

Oxygen was applied for 20–40 sec immediately after TBI. Then, the scalp wound was closed with a polypropylene 6-0 suture (Surgipro^TM^ II, Covidien, Mansfield, MA), and the mice were returned to their home cages with free access to water and food. Sham animals underwent the same procedures as the animals in the TBI group, but without the release of the weight.

### Neurological severity score (NSS)

Behavioral testing was performed by experienced scientists blinded to the experimental group. The neurobehavioral status of the mice was obtained by the NSS.^5^ The general neurological state of the mice was evaluated at baseline and 2 h and 24 h post-injury during the light part of the light–dark cycle. The NSS consists of nine individual parameters, including motor function, alertness, and physiological behavior tasks.

The following items were assessed: presence of paresis; impairment of seeking behavior; absence of perceptible startle reflex; inability to get down from a rectangle platform (34 ** ×** 27 cm); inability to walk on 3-, 2-, and 1-cm wide beams; and inability to balance on a 0.7-cm–wide beam and a 0.5 cm-diameter round beam for at least 15 sec. If a mouse showed impairment in one of these items, then a value of 1 was added to its NSS. Thus, higher values for the NSS indicate more severe neurological impairment.

The severity of skull fractures was determined based on the parameters used to evaluate skull fractures in patients with small modifications: non (skull bone without changes/no signs of fracture), mild (isolated linear fracture with no evidence of intracranial lesion), moderate (linear depressed or diastatic fracture with minimal dural tear, no macroscopic evidence of hematoma or parenchyma injury), and severe (complicated fracture with macroscopic intracranial lesions, including parenchyma injury and hematomas).^27,28^

The severity of skull fracture was evaluated immediately after injury by macroscopic visualization, which is a rapid and simple method. Post-mortem micro-CT analysis of the parietal bone plates could provide more detailed results than macroscopic visualization, however.

### Open-field test

To detect the motor activity of the animals, an open-field test was performed before and at 12 h and 1, 3, and 14 days after TBI. The test apparatus was a square arena (44 × 44 cm) with a black floor. The moved distance and velocity in a 5-min session were recorded and analyzed using the EthoVision video tracking system (version XT 11.5; Noldus, Wageningen, The Netherlands).

### Quantitative RT-PCR analysis

To quantify the inflammatory response, the mouse brains were collected at 12 h and 1, 3, and 14 days after trauma. Brain tissues were collected immediately after decapitation of the animals. Individual brain structures from both hemispheres were separated on ice and snap frozen in liquid nitrogen. The brain samples were stored at -80°C until ribonucleic acid (RNA) isolation.

Quantitative RT-PCR was performed using a previously described method^29^ with slight modifications. Total RNA was isolated from brain tissues using an RNA mini kit (Life Technologies, Grand Island, NY) according to the manufacturer's instructions. The extracted RNA was dissolved in 50 μL nuclease-free distilled water and stored at -80°C until further analysis. First-strand complementary deoxyribonucleic acid (cDNA) was synthesized using a high-capacity cDNA reverse transcription kit (Thermo Fisher Scientific, Waltham, MA) following the manufacturer's protocol.

Quantitative RT-PCR analysis for IL-6, IL-1β, TNF-α, tissue inhibitor of metalloproteinase (TIMP)-1, matrix metalloproteinase (MMP)-9 and β-actin was performed using SYBR^®^ Green Master Mix (Life Technologies). The primer sequences used in this study were as follows: IL-6 (NM_001314054.1), 5 - TCT ATA CCA CTT CAC AAG TCG GA - 3 (forward) and 5 – GAA TTG CCA TTG CAC AAC TCT TT – 3 (reverse); IL-1β (NM_008361.4), 5 – GGG CCT CAA AGG AAA GAA TC – 3 (forward) and 5 – TTG CTT GGG ATC CAC ACT CT – 3 (reverse); TNF-α (NM_013693.3), 5 – CCC TCA CAC TCA GAT CAT CTT CT – 3 (forward) and 5 – GCT ACG ACG TGG GCT ACA G – 3 (reverse); TIMP-1 (NM_011593.2), 5 – CAG TAA GGC CTG TAG CTG TGC – 3 (forward) and 5 - AGG TGG TCT CGT TGA TTT CTG – 3 (reverse); MMP-9 (NM_031055.1), 5 – TCG AAG GCG ACC TCA AGT G – 3 (forward) and 5 – TTC GGT GTA GCT TTG GAT CCA – 3 (reverse); and β-actin (NM_007393.5), 5 – CCT CTA TGC CAA CAC AGT GC– 3 (forward) and 5 – CAT CGT ACT CCT GCT TGC TG– 3 (reverse).

The primers were obtained from Metabion (Steinkirchen, Germany). The relative expression levels for each gene were calculated with the ΔΔCt method, normalized to the expression of β-actin and compared with the age-matched sham group.

### TIMP-1 and TNF-α measurement in plasma

Blood samples were collected after decapitation of the animals. Heparin was used to prevent blood clot formation. Blood samples were centrifuged at 3000 rpm and 4°C for 10 min (Heraeus™ Biofuge™ Stratos™ Centrifuge, Thermo Fisher Scientific) to separate the plasma. Plasma was stored at -80°C. The ELISA was performed using Mouse TIMP1 SimpleStep ELISA^®^ Kit (Abcam, Boston, MA) and a Mouse TNF-α ELISA Kit (EMD Millipore, Burlington, MA) following the manufacturer's instructions.

### Micro-CT analysis

The micro-CT imaging was performed before TBI. The mice were anesthetized using 4% isoflurane in a mixture of 50% nitrous oxide and 50% oxygen, followed by 1–1.5% isoflurane throughout the procedure. The micro-CT scans were acquired using 30 keV, 0.95 mA lamp energy with an exposure time of 250 ms using a Trifoil InSyTe FLECT^®^ imager (Chatsworth, CA). A total of 720 projections were acquired per rotation. Three-dimensional images on selected skull areas were reconstructed with a voxel size of 25 μm using FluoroView 1.5 and Cobra 7.12.9 software. Qualitative and quantitative data were analyzed using VivoQuant 1.23 software.

To evaluate parietal bone thickness and density, a coronal section was taken. The frontal plane represents the coronal plane at the middle of the bregma and lambda sutures. By using coronal sections, the parietal bone gray scale value (radiological density) was measured in three regions (Fig. 6B) of interest at five points in an equal interval. The coronal skull micro-CT images were divided into bone and non-bone regions by determining the threshold value using the automated Otsu method.^30^ The gray scale values of the mouse skull were used to evaluate bone density.^31–33^

### Measurement of contusion surface area

Mice were anesthetized using a combination of ketamine (200 mg/kg) and xylazine (15 mg/kg) and perfused transcardially with 0.01 M phosphate-buffered saline (1X PBS) for 5 min at a speed of 3 mL/min to remove blood from the tissue. Perfusion was switched to 4% paraformaldehyde (PFA) solution in 1X PBS until stiffening of the mouse body. The brains were dissected carefully and fixed overnight in 4% PFA at 4°C. The dissected brains were washed in 1X PBS, dried, and photographed using a digital camera (Sony A900, Japan). The cortical contusion area was quantified using ImageJ software (version 1.52j).

### Statistical analysis

The statistical calculations were performed using the GraphPad Prism 8.1 software package (GraphPad Software, Inc., La Jolla, CA). The Shapiro-Wilk test was used to examine the distribution of the data. The Kruskal-Wallis test followed by the Dunn test was used for non-normally distributed data sets. A *post hoc* test was performed if analysis of variance (ANOVA) or the Kruskal-Wallis test indicated statistically significant differences.

Time and group interactions were analyzed by using two-way ANOVA and the Tukey test as the *post hoc* test for multiple comparisons. Student *t* test was used to compare differences between mouse weight and occurrence of fracture and differences between parietal bone thickness and gray scale values in animals without and with skull fractures. The Pearson correlation test was used to analyze the correlation between parietal bone thickness and animal weight. The *p* values less than 0.05 were considered significant.

## Results

### Health status assessment after CHI

Two mice in the “with fracture” group died immediately after injury. All other mice were included in the data analysis. One mouse in the “with fracture” group had spontaneous seizures. In animals with skull fracture, there was a minor loss of body weight during the first three post-injury days; however, no significant differences were observed between the groups (data not shown). During the immediate time after injury, apnea (<3 sec) was observed in three animals. The remaining animals breathed spontaneously during and after the procedure and were fully awake within 1 min.

### NSS

**FIG. 2.**
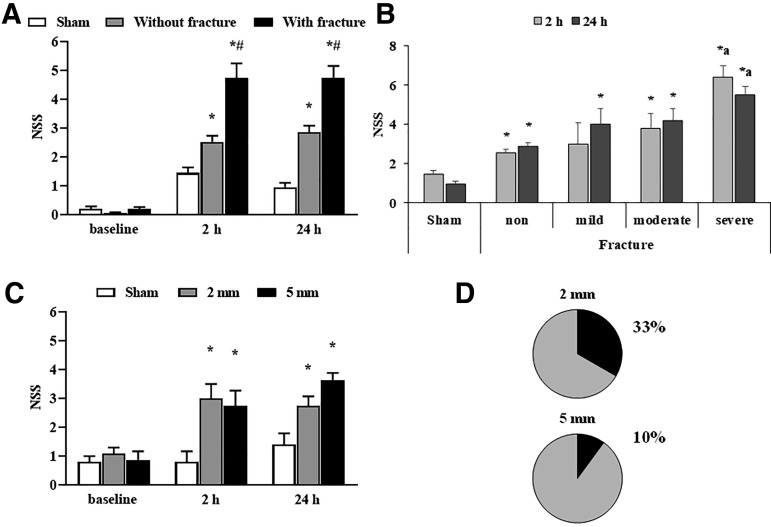
Functional outcome after traumatic brain injury (TBI). (**A**) Comparison of the neurobehavioral responses in animals without or with skull fractures using the Neurological Severity Score (NSS). Data are shown as the mean ± standard error of the mean (SEM). (sham-operated, *n* = 24; without fracture, *n* = 52; with fracture, *n* = 33). *p < 0.05 vs. sham-operated; ^#^*p* < 0.05 without vs. with skull fracture (repeated measures two-way analysis of variance (ANOVA) followed by the Tukey test). (**B**) Relation of the severity of skull fracture and NSS at 2 h and 24 h after TBI. Data are shown as the mean with 95% confidence interval (sham-operated, *n* = 24; non-fracture, *n* = 52; mild fracture, *n* = 7; moderate fracture, *n* = 12; severe fracture, *n* = 14). *p < 0.05 vs. sham-operated; ^a^*p* < 0.05 non vs. severe fracture (Kruskal Wallis test followed by the Dunn test). (**C**) NSS after TBI using 2 mm and 5 mm tips in animals without skull fractures. Data are shown as the mean ± SEM. *p < 0.05 vs. sham-operated (repeated measures two-way ANOVA followed by the Tukey test) and (**D**) occurrence of fractures using cone with a 2 mm or 5 mm diameter tip (sham-operated, *n* = 5; 2 mm, *n* = 12; 5 mm, *n* = 8).

The animals were divided into two groups: animals with and without skull fractures after TBI. The NSS was significantly higher in traumatized than in sham-operated mice at 2 h and 24 h after surgery (Fig. 2A; main effects of time [F_(2, 135)_ = 98.4, *p* < 0.0001] and group [F_(2, 76)_ = 25.8, *p* < 0.0001], and interaction between time and group [F_(4, 152)_ = 26.9, *p* < 0.0001]).

Two hours after TBI, the average scores for animals without and with skull fractures were 2.5 ± 0.2 and 4.8 ± 0.5 points, respectively (Fig. 2A). Twenty-four hours after TBI, the NSS for animals without and with skull fracture was 2.9 ± 0.2 and 4.8 ± 0.4 points, respectively (Fig. 2A), and there was a significant difference between the groups (Fig. 2A). In addition, we observed that the NSS increased significantly depending on the skull fracture severity (Fig. 2B).

We compared the impact of cone tips on the occurrence of fracture using a cone with a 2 mm or 5 mm tip. The cone with a 2 mm tip caused skull fractures in 33% of animals, while only 10% of animals had fractures when traumatized with the 5 mm cone (Fig. 2D). The functional outcome was similar in mice traumatized with the 2 or 5 mm cone at 2 h and 24 h after TBI (Fig. 2C; main effects of time [F_(2, 34)_ = 18.8, *p* < 0.0001] and group [F_(2, 22)_ = 5.3, *p* < 0.05] and the interaction between time and group [F_(4, 44)_ = 3.4, *p* < 0.05]).

### Open-field test

The mice were tested in an open-field test to assess locomotor activity at 12 h, 1, 3, and 14 days after injury. There were no significant differences in the distance traveled and velocity between groups (data not shown).

### Inflammation-related gene expression in the brain after TBI

**FIG. 3.**
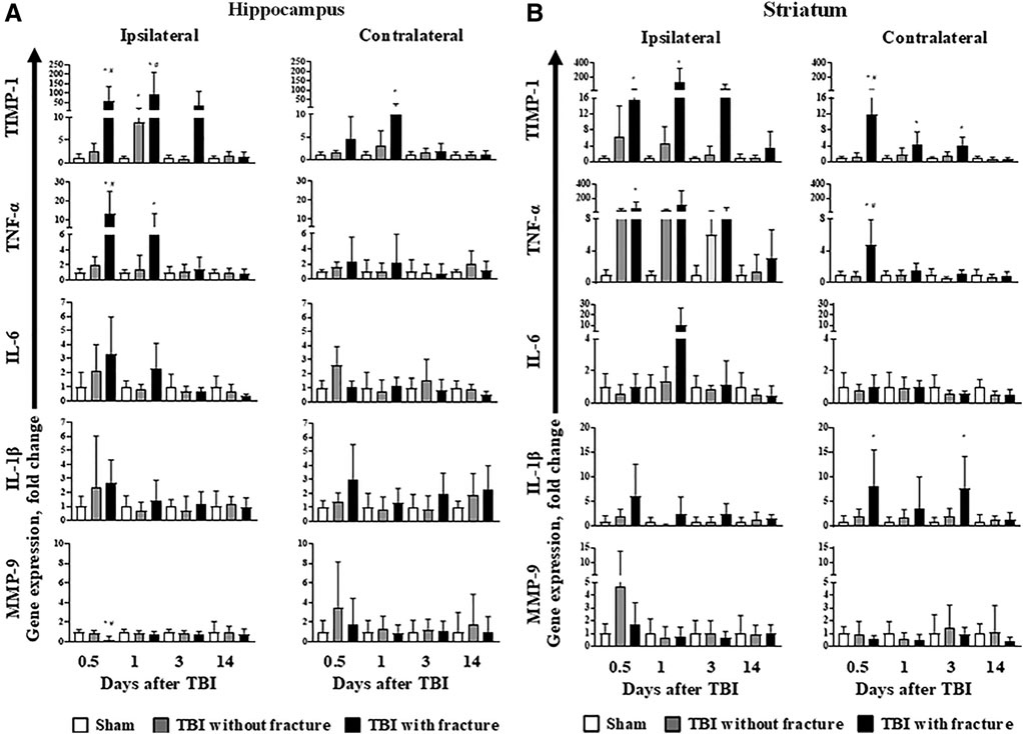
Inflammation-related gene expression in the ipsilateral and contralateral (**A**) hippocampus and (**B**) striatum after traumatic brain injury (TBI). Data are expressed as the mean with 95% confidence interval (*n* = 5–9). *p < 0.05 vs. sham-operated; ^#^*p* < 0.05 TBI without vs. TBI with skull fracture (ordinary two-way analysis of variance followed by the Tukey test; see Supplementary Table S1 for F values, degrees of freedom, and *p* values). TIMP-1, tissue inhibitor of metalloproteinase-1; TNF-α, tumor necrosis factor-α ;IL, interleukin; MMP-9, matrixmetalloproteinase-9.

To detect the impact of skull fracture on inflammatory gene expression in brain tissue, we measured IL-6, IL-1β, TNF-α, MMP-9, and TIMP-1 gene expression in the hippocampus and striatum at 12 h, 1, 3, and 14 days after TBI. For gene expression data F values, degrees of freedom and *p* values are summarized in Supplementary Table S1. A significant 71- and 90-fold increase was observed for TIMP-1 at 12 h and 1 day after injury, respectively, in the ipsilateral hippocampus of animals with skull fractures (Fig. 3A). In addition, TIMP-1 was increased in the contralateral hippocampus 1 day after injury (Fig. 3A).

The TIMP-1 mRNA expression in the ipsilateral striatum of animals with skull fractures was increased 16- and 130-fold at 12 h and 1 day after injury, respectively (Fig. 3B). In addition, TIMP-1 mRNA was increased in the contralateral striatum at 1 and 3 days after injury (Fig. 3B).

The TNF-α mRNA expression in animals with skull fractures was significantly increased 13-fold and six-fold in the ipsilateral hippocampus at 12 h and 1 day after injury, respectively (Fig. 3A). The TNF-α mRNA expression was significantly increased 67-fold and five-fold in the ipsilateral and contralateral striatum, respectively, in animals with skull fractures at 12 h after injury (Fig. 3B). The MMP-9 gene was significantly downregulated at 12 h after trauma in the ipsilateral hippocampus of animals with skull fractures (Fig. 3A). No differences in IL-6 gene expression were detected between groups in the hippocampus and striatum (Fig. 3A, B).

Significant increase was observed for TIMP-1 mRNA at 1 day after injury in the ipsilateral hippocampus of animals without skull fractures (Fig. 3A). No significant changes for IL-6, IL-1β, TNF-α, and MMP-9 gene expression were observed at 12 h and 1, 3, and 14 days after TBI in animals without skull fractures (Fig. 3A, B).

**FIG. 4.**
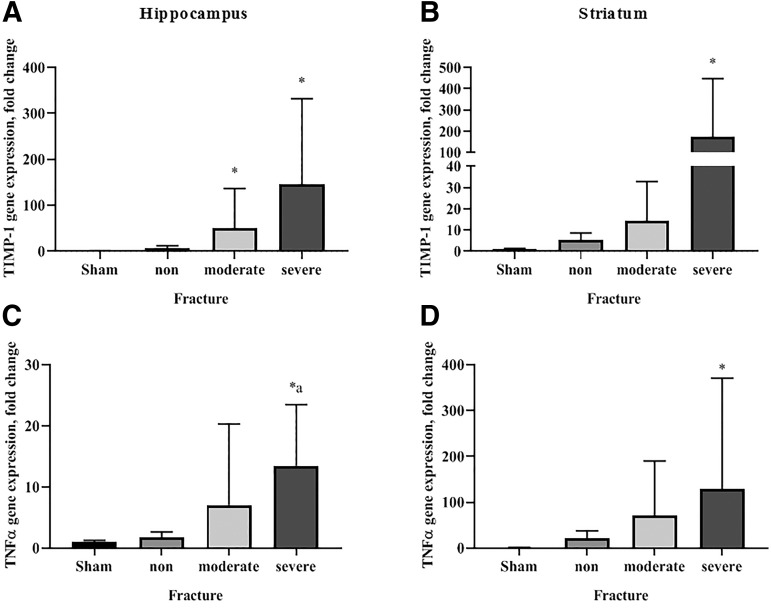
Inflammation-related gene expression in the ipsilateral brain at 12 h and 24 h after traumatic brain injury with various severities of skull fracture. Tissue inhibitor of metalloproteinase-1 (TIMP-1) gene expression in the hippocampus (**A**) and striatum (**B**) and tumor necrosis factor-α (TNF-α) gene expression in the hippocampus (**C**) and striatum (**D**). Data are expressed as the mean with 95% confidence interval (*n* = 5–13). *p < 0.05 vs. sham-operated; ^a^*p* < 0.05 vs. non-fracture; ^b^*p* < 0.05 vs. mild fracture; ^c^*p* < 0.05 vs. moderate fracture (Kruskal-Wallis test followed by the Dunn test).

The TIMP-1 and TNF-α genes showed a significant correlation with the severity of TBI at 12 h and 24 h after injury (Fig. 4). In the ipsilateral hippocampus, 50- and 146-fold increases in TIMP-1 gene expression were observed after moderate and severe skull fractures, respectively (Fig. 4A). Similarly, 14- and 173-fold increases in TIMP-1 gene expression in the ipsilateral striatum were observed after moderate and severe skull fractures, respectively (Fig. 4B). After severe skull fracture, TNF-α mRNA showed 14- and 130-fold increases in the ipsilateral hippocampus and striatum, respectively (Fig. 4C and 4D).

### TIMP-1 and TNF-α levels in plasma

**FIG. 5.**
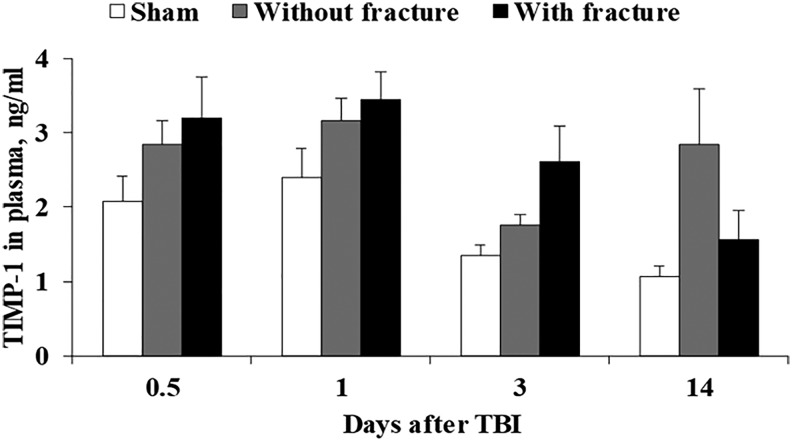
Tissue inhibitor of metalloproteinase-1 (TIMP-1) concentration in the plasma of animals without or with skull fractures at 12 h, 1, 3, and 14 days after traumatic brain injury (TBI). Data are expressed as the mean ± standard deviation (n = 5–7). *p < 0.05 vs. sham-operated (ordinary two-way analysis of variance followed by the Tukey test).

The TIMP-1 and TNF-α protein concentrations were measured in plasma at 12 h and 1, 3, and 14 days after TBI. Slightly increased TIMP-1 plasma levels were observed in animals with fractures (Fig. 5; main effects of time [F_(3, 53)_ = 6.0, *p* < 0.01] and group [F_(2, 53)_ = 6.6, *p* < 0.01] and interaction between time and group [F_(6, 53)_ = 1.9, *p* > 0.05]); however, there were no significant differences when compared with sham-operated animals. Plasma levels of TNF-α were not elevated after TBI (<1.5 pg/mL).

### Evaluation of the skull bone by micro-CT

**FIG. 6.**
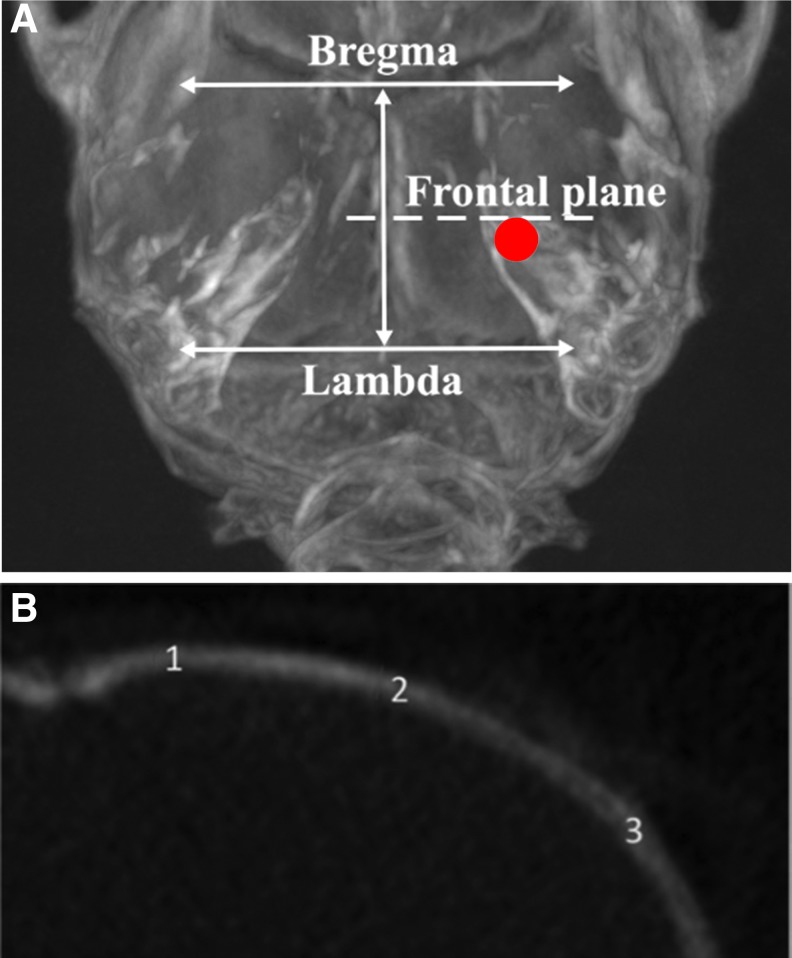
Micro-CT image of a mouse skull with schematic illustration of the measurement parameters of mouse parietal bone thickness and density. (**A**) Frontal plane at a distance between coronal suture and lambdoid suture, which indicates the impact location (red circle) in the weight-drop traumatic brain injury model. (**B**) The landmarks shown on a coronal section of parietal bone represent thickness and density measurement points (medial periphery–1, midline–2, and lateral periphery–3). Color image is available online.

Micro-CT examination of the parietal bone revealed variations in thickness and gray scale values. The average parietal bone thickness varied from 0.17 to 0.44 mm (mean, 0.3 ± 0.1 mm), depending on the localization. The parietal bone became thinner as it reached the sagittal suture (Fig. 6B). The lateral periphery of the parietal bone had a thicker structure with a higher gray scale value, ranging from 3.7 to 10.2 (mean, 6.7 ± 1.7). The parietal bone gray scale value was higher in 20-week-old mice than in 10-week-old mice (mean, 7.2 vs. 7.5; 10 weeks vs. 20 weeks, respectively). Lower parietal bone gray scale values were associated significantly with skull fracture.

**FIG. 7.**
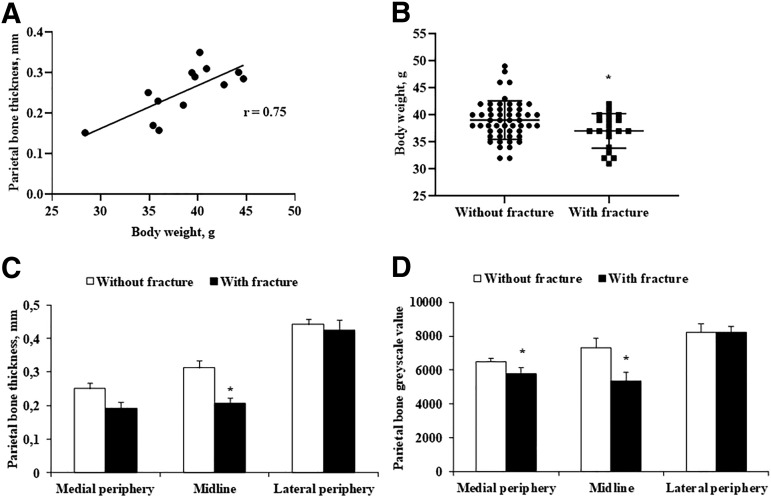
Correlation between parietal bone thickness, mouse body weight and skull fracture. (**A**) Midline parietal bone thickness as a function of animal body weight (*n* = 13). Parietal bone thickness was measured using a Trifoil InSyTe FLECT^®^ imager (Pearson *r = 0.748*, *p < 0.01). (**B**) Difference between mouse weight and occurrence of fracture. Data were analyzed using GraphPad Prism 8.1 software (without fracture, *n* = 52; with fracture, *n* = 20). *p < 0.05 vs. group without fracture (Student *t* test). (**C**) Difference between parietal bone thickness and **(D)** gray scale values in animals without and with skull fracture. Parietal bone thickness and gray scale values were measured at three points using a Trifoil InSyTe FLECT^®^ imager. Data are shown as the mean ± standard error of the mean of five animals. *p < 0.05 without vs. with fracture (Student *t* test).

Skull thickness increased proportionally with age and weight of the animals. Parietal bone thickness correlated with animal weight (Pearson *r* = 0.75, *p* < 0.01), and animal weight was inversely associated with skull fractures. Decreased parietal bone thickness and gray scale values were significantly associated with an increased risk of fracture (Fig. 7).

### Evaluation of the contusion surface area

**FIG. 8.**
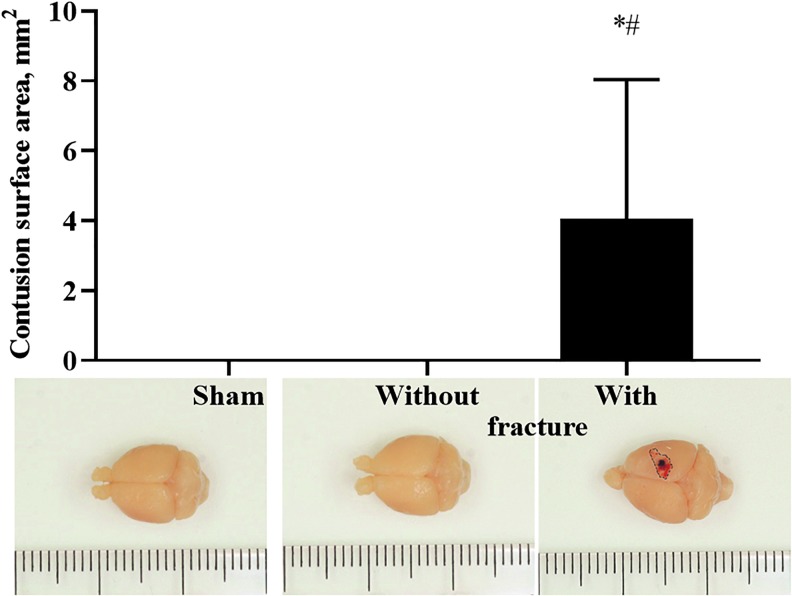
Average contusion surface area observed up to 3 days after traumatic brain injury. Data are shown as the mean ± 95% confidence interval. (sham-operated, *n* = 6, without fracture, *n* = 6, with fracture, *n* = 7). Representative images of brains are shown under the graph. *p < 0.05 vs. sham-operated; ^#^*p* < 0.05 vs. without fracture (Kruskal-Wallis test followed by the Dunn test). Color image are available online.

Macroscopic observation demonstrated that the brains of sham-operated mice and mice that underwent TBI without skull fracture showed no signs of cortical injury (Fig. 8). The TBI with skull fracture resulted in mild to severe brain damage because of skull depression (Fig. 8). In addition, skull fractures were associated with epidural and subdural hematomas and intraparenchymal hemorrhage.

## Discussion

A TBI is a complex and heterogeneous injury that includes hematomas, contusions, subarachnoid hemorrhage, hypoxia, ischemia, and axonal and vascular injuries. This heterogeneity is a particular challenge when performing experimental TBI research.^12^ The WD model is the most commonly used experimental method to study the pathophysiology of CHI.^4^ Nevertheless, this model is not well standardized and generates results with substantial variability regarding histopathological, biochemical, and functional outcomes.^7–11,15,28,36–38^ Our results demonstrated that animals experiencing skull fractures after undergoing the WD model show a substantial inflammatory response in the brain, while animals without skull fractures do not, thus demonstrating how skull fractures contribute to the heterogeneity of the WD model.

When reviewing published literature (articles published on the CHI model in PubMed from 2001 to 2018), skull fractures were evaluated in only 18 of 97 studies (18.6%), and animals with fractures were excluded. A significant number of studies (81.4%) did not mention the incidence of skull fractures, showing that injury to the skull has been neglected in most CHI experiments.

Moreover, significant heterogeneity was observed in the parameters of falling weight, drop height, and impact tip diameter that led to different impact energies delivered to brain tissue. The falling weight ranged from 5 to 500 g, and the drop height ranged from 1 to 167 cm. Impact tip diameters ranged from 1 to 5 mm or were not reported. The calculated amount of energy as a function of the mass and height varied from 0.04 to 1.10 J. Thus, these studies do not share a common methodological indicator that would allow a comparison of the results of experiments between laboratories.

Our current study investigating the influence of skull fractures after WD represents a first step to reduce heterogeneity in this very useful CHI model. Skull fracture is an independent risk factor for intracranial hemorrhage in patients with TBI.^34,35^ Intracranial hemorrhage is associated with increased intracranial pressure, oxidative damage, vasogenic edema, cytotoxic edema, heme toxicity, and iron toxicity and may thus contribute to secondary brain injury.^36^

Approximately one-half of depressed skull fractures in patients with TBI result in dural tearing, which is associated with cranial infections.^37,38^ In patients, skull fracture with hemorrhage results in increased inflammation and neuronal excitability because of the toxic effects of hemoglobin breakdown and the generation of reactive oxygen species.^39^

Thus, our results demonstrating an increased neuroinflammatory response in the brain after TBI with skull fracture are well in line with the clinical features of TBI. Moreover, compared with animals without fracture, TBI in animals with moderate and severe fractures showed significant differences in responses to the NSS at 24 h but not at 2 h after TBI. Only one study reported animals with and without skull fractures and divided these animals into separate groups.^40^ There was no difference in NSS between animals with or without skull fracture and sham-operated animals at 1 h post-injury.^40^ The NSS was not assessed at later time points after an injury; thus, it is difficult to compare the impact of fracture on NSS in this study. Moreover, skull fracture was associated with more severe TBI outcomes, including immediate post-traumatic respiratory depression, secondary rebound injury, and death in mice.^6^

Other important factors, such as age and skull thickness, could also influence the incidence of parietal bone fracture. Skull cortical thickness is an important factor related to the deformation of the skull and fracture propensity,^41^ and skull cortical bone density also influences skull fracture in humans.^42^ Moreover, skull density is associated with the biomechanical threshold for concussion and may influence TBI, even without causing a skull fracture.^43^ We showed that mouse parietal bone thickness and gray scale values are effective at predicting skull fractures. Thus, mouse age and weight should be taken into account in the design of the experiment.

It is important to acknowledge that in other CHI models, helmets (i.e., metallic disc on the exposed skull surface) are used to reduce the incidence of skull fractures.^7,9,44^ The use of helmets helps to disperse the impact energy delivered to the brain tissue, thereby protecting the skull from the fractures. In the present study, the use of a 5 mm cone tip reduced the incidence of skull fractures, suggesting that a larger diameter should be used in the WD model.

To date, contrasting results on the neuroinflammatory response in brain tissues have been reported after CHI in mice. Our results clearly showed a significant increase in TNF-α and TIMP-1 gene expression in animals with skull fractures at 12 h and 24 h after injury, while gene expression was unchanged in animals without fracture. We did not observe a difference in the TIMP-1 plasma concentration between animals with or without fractures, however.

Many studies have demonstrated a significantly increased neuroinflammatory response in brain tissue, reaching levels greater than physiological levels within hours of injury^19,45–48^; however, only one study did not observe skull fractures.^48^ For example, significantly increased brain levels of TNF-α gene and protein expression were observed on the first day after trauma,^19,47,49^ while no changes in TNF-α expression were observed in other studies.^17,18,50^ Interestingly, a significant increase in TNF-α expression at 4 h and 48 h after WD injury was observed using only animals with skull fractures in a WD model.^49^

Moreover, there is a significant increase in IL-1, IL-6, IL-10, and TNF-α cytokines in response to craniotomy *per se* compared with those in naïve animals,^51,52^ indicating that skull fractures could cause inflammation and create outcome heterogeneity within the groups in the WD model. Recent studies found that combined closed-skull TBI and tibial fractures worsened outcome and increased inflammation compared with mice given only a TBI.^53,54^ Similar to our findings, the levels of inflammatory cytokines, such as IL-1β, IL-6, and TNF-α, were not increased in isolated-TBI.^53^ Interestingly, in a recent study, muscle injury combined with TBI did not significantly impact inflammatory cytokine expression or functional outcome after WD-TBI,^55^ suggesting that a concurrent fracture rather than soft tissue injury may exacerbate neuroinflammation and worsen the outcomes after TBI.

Previous research demonstrated that the overexpression of TNF-α was correlated with the severity of trauma in fluid-percussion–induced TBI.^45^ We observed a correlation between the levels of TNF-α and TIMP-1 gene expression and the severity of fracture. Moreover, TIMP-1 gene expression was elevated significantly in the ipsilateral hippocampus and striatum during the first 3 days after trauma with skull fracture, showing an increase of more than 100-fold.

Previous research identified an association between serum TIMP-1 levels and TBI severity and death.^56^ A previous study demonstrated that the TIMP-1 gene is overexpressed in the brain at 12 h after middle cerebral artery occlusion, reaching a peak level at 2 days after stroke.^57,58^ High serum TIMP-1 levels are also associated with a worse prognosis after stroke.^56,59^ The TIMP-1 is constitutively expressed at a low level in many tissues, but after tissue injury and inflammation, TIMP-1 gene expression generally increases compared with that in healthy tissue.^60,61^

Pro-inflammatory cytokines increase the expression of TIMP-1 in the brain.^62^ In the present study, high TIMP-1 inflammatory gene expression was observed in the first 3 days after TBI with skull fracture compared with the gene expression levels at 14 days after TBI, showing an acute response to injury. Because severe skull fracture was observed together with skull depression and bleeding, the high TIMP-1 gene expression in the brain was probably a rapid and acute response to the massive infiltration of proinflammatory cytokines that occurred in the brain after dural injury and bleeding.

## Conclusion

Compared with TBI without fractures, TBI with skull fractures resulted in a more severe neurobehavioral response and a considerable increase in inflammatory gene expression in brain tissue. Our data suggest that when using the WD model, the data from animals with skull fractures must be analyzed separately from those without skull fractures to reduce the heterogeneity of the model. Heterogeneity may also be reduced *a priori* by using animals with a thicker skull bone—i.e., older mice—and by using an impact cone with a larger diameter—i.e., 5 mm instead of 2 mm—or helmets—i.e., metallic disc—on the skull surface.

## Supplementary Material

Supplemental data

## References

[1] De Guzman E. and Ament A. (2017). Neurobehavioral management of traumatic brain injury in the critical care setting: an update. Crit. Care Clin. 33, 423–440 28601130 10.1016/j.ccc.2017.03.011

[2] Berkner J., Mannix R., and Qiu J. (2016). Clinical traumatic brain injury in the preclinical setting. Methods Mol. Biol. 1462, 11–28 27604710 10.1007/978-1-4939-3816-2_2

[3] Kerr H.A. (2013). Closed head injury. Clin. Sports Med. 32, 273–287 23522509 10.1016/j.csm.2012.12.008

[4] Namjoshi D.R., Good C., Cheng W.H., Panenka W., Richards D., Cripton P.A., and Wellington C.L. (2013). Towards clinical management of traumatic brain injury: a review of models and mechanisms from a biomechanical perspective. Dis. Model. Mech. 6, 1325–1338 24046354 10.1242/dmm.011320 PMC3820257

[5] Albert-Weißenberger C., Várrallyay C., Raslan F., Kleinschnitz C., and Sirén A.-L. (2012). An experimental protocol for mimicking pathomechanisms of traumatic brain injury in mice. Exp. Transl. Stroke Med. 4, 1 22300472 10.1186/2040-7378-4-1 PMC3305492

[6] Flierl M.A., Stahel P.F., Beauchamp K.M., Morgan S.J., Smith W.R., and Shohami E. (2009). Mouse closed head injury model induced by a weight-drop device. Nat. Protoc. 4, 1328–1337 19713954 10.1038/nprot.2009.148

[7] Marmarou A., Foda M.A.A.-E., Brink W. van den Campbell, J., Kita H., and Demetriadou K. (1994). A new model of diffuse brain injury in rats. Part 1: pathophysiolpgy and biomechanics. J. Neurosurg. 80, 291–300 8283269 10.3171/jns.1994.80.2.0291

[8] Namjoshi D.R., Cheng W., McInnes K.A., Martens K.M., Carr M., Wilkinson A., Fan J., Robert J., Hayat A., Cripton P.A., and Wellington C.L. (2014). Merging pathology with biomechanics using CHIMERA (Closed-Head Impact Model of Engineered Rotational Acceleration): a novel, surgery-free model of traumatic brain injury. Mol. Neurodegener. 9, 55 25443413 10.1186/1750-1326-9-55 PMC4269957

[9] Meconi A., Wortman R.C., Wright D.K., Neale K.J., Clarkson M., Shultz S.R., and Christie B.R. (2018). Repeated mild traumatic brain injury can cause acute neurologic impairment without overt structural damage in juvenile rats. PLoS One 13, e0197187 29738554 10.1371/journal.pone.0197187 PMC5940222

[10] Ren Z., Iliff J.J., Yang L., Yang J., Chen X., Chen M.J., Giese R.N., Wang B., Shi X., and Nedergaard M. (2013). ‘Hit & Run’ model of closed-skull traumatic brain injury (TBI) reveals complex patterns of post-traumatic AQP4 dysregulation. J. Cereb. Blood Flow Metab. 33, 834–845 23443171 10.1038/jcbfm.2013.30 PMC3677112

[11] Cernak I., Merkle A.C., Koliatsos V.E., Bilik J.M., Luong Q.T., Mahota T.M., Xu L., Slack N., Windle D., and Ahmed F.A. (2011). The pathobiology of blast injuries and blast-induced neurotrauma as identified using a new experimental model of injury in mice. Neurobiol. Dis. 41, 538–551 21074615 10.1016/j.nbd.2010.10.025

[12] Xiong Y., Mahmood A., and Chopp M. (2013). Animal models of traumatic brain injury. Nat. Rev. Neurosci. 14, 128–142 23329160 10.1038/nrn3407 PMC3951995

[13] Kane M.J., Angoa-Pérez M., Briggs D.I., Viano D.C., Kreipke C.W., and Kuhn D.M. (2012). A mouse model of human repetitive mild traumatic brain injury. J. Neurosci. Methods 203, 41–49 21930157 10.1016/j.jneumeth.2011.09.003 PMC3221913

[14] Wu Q., Xuan W., Ando T., Xu T., Huang L., Huang Y.-Y., Dai T., Dhital S., Sharma S.K., Whalen M.J., and Hamblin M.R. (2012). Low-level laser therapy for closed-head traumatic brain injury in mice: effect of different wavelengths. Lasers Surg. Med. 44, 218–226 22275301 10.1002/lsm.22003 PMC3397203

[15] Schwarzbold M.L., Rial D., De Bem T., Machado D.G., Cunha M.P., dos Santos A.A., dos Santos D.B., Figueiredo C.P., Farina M., Goldfeder E.M., Rodrigues A.L.S., Prediger R.D.S., and Walz R. (2010). Effects of traumatic brain injury of different severities on emotional, cognitive, and oxidative stress-related parameters in mice. J. Neurotrauma 27, 1883–1893 20649482 10.1089/neu.2010.1318

[16] Midura E.F., Jernigan P.L., Kuethe J.W., Friend L.A., Veile R., Makley A.T., Caldwell C.C., and Goodman M.D. (2015). Microparticles impact coagulation after traumatic brain injury. J. Surg. Res. 197, 25–31 25846728 10.1016/j.jss.2015.02.064 PMC5857955

[17] Chhor V., Moretti R., Le Charpentier T., Sigaut S., Lebon S., Schwendimann L., Oré M.V., Zuiani C., Milan V., Josserand J., Vontell R., Pansiot J., Degos V., Ikonomidou C., Titomanlio L., Hagberg H., Gressens P., and Fleiss B. (2017). Role of microglia in a mouse model of paediatric traumatic brain injury. Brain. Behav. Immun. 63, 197–209 27818218 10.1016/j.bbi.2016.11.001 PMC5441571

[18] Albert-Weissenberger C., Stetter C., Meuth S.G., Göbel K., Bader M., Sirén A.L., and Kleinschnitz C. (2012). Blocking of bradykinin receptor B1 protects from focal closed head injury in mice by reducing axonal damage and astroglia activation. J. Cereb. Blood Flow Metab. 32, 1747–1756 22569191 10.1038/jcbfm.2012.62 PMC3434625

[19] Baratz R., Tweedie D., Wang J.-Y., Rubovitch V., Luo W., Hoffer B.J., Greig N.H., and Pick C.G. (2015). Transiently lowering tumor necrosis factor-α synthesis ameliorates neuronal cell loss and cognitive impairments induced by minimal traumatic brain injury in mice. J. Neuroinflammation 12, 45 25879458 10.1186/s12974-015-0237-4 PMC4352276

[20] Gyoneva S., and Ransohoff R.M. (2015). Inflammatory reaction after traumatic brain injury: therapeutic potential of targeting cell–cell communication by chemokines. Trends Pharmacol. Sci. 36, 471–480 25979813 10.1016/j.tips.2015.04.003 PMC4485943

[21] Zhang Q., Zhou C., Hamblin M.R., and Wu M.X. (2014). Low-level laser therapy effectively prevents secondary brain injury induced by immediate early responsive gene X-1 deficiency. J. Cereb. Blood Flow Metab. 34, 1391–1401 24849666 10.1038/jcbfm.2014.95 PMC4126101

[22] Clausen F., Marklund N., and Hillered L. (2019). Acute inflammatory biomarker responses to diffuse traumatic brain injury in the rat monitored by a novel microdialysis technique. J. Neurotrauma 36, 201–211 29790398 10.1089/neu.2018.5636

[23] Rowe R.K., Ellis G.I., Harrison J.L., Bachstetter A.D., Corder G.F., Van Eldik L.J., Taylor B.K., Marti F., and Lifshitz J. (2016). Diffuse traumatic brain injury induces prolonged immune dysregulation and potentiates hyperalgesia following a peripheral immune challenge. Mol. Pain 12, 174480691664705 10.1177/1744806916647055 PMC4955995 27178244

[24] Garg C., Seo J.H., Ramachandran J., Loh J.M., Calderon F., and Contreras J.E. (2018). Trovafloxacin attenuates neuroinflammation and improves outcome after traumatic brain injury in mice. J. Neuroinflammation 15, 42 29439712 10.1186/s12974-018-1069-9 PMC5812039

[25] Kilkenny C., Browne W., Cuthill I.C., Emerson M., Altman D.G., and NC3Rs Reporting Guidelines Working Group. (2010). Animal research: reporting in vivo experiments: the ARRIVE guidelines. Br. J. Pharmacol. 160, 1577–1579 20649561 10.1111/j.1476-5381.2010.00872.x PMC2936830

[26] McGrath J., Drummond G., McLachlan E., Kilkenny C., and Wainwright C. (2010). Guidelines for reporting experiments involving animals: the ARRIVE guidelines. Br. J. Pharmacol. 160, 1573–1576 20649560 10.1111/j.1476-5381.2010.00873.x PMC2936829

[27] Powell E.C., Atabaki S.M., Wootton-Gorges S., Wisner D., Mahajan P., Glass T., Miskin M., Stanley R.M., Jacobs E., Dayan P.S., Holmes J.F., and Kuppermann N. (2015). Isolated linear skull fractures in children with blunt head trauma. Pediatrics 135, e851–e857 25780067 10.1542/peds.2014-2858

[28] McGrath A. and Taylor R.S. (2019). Pediatric Skull Fractures. StatPearls Publishing 29489156

[29] Dambrova M., Zvejniece L., Skapare E., Vilskersts R., Svalbe B., Baumane L., Muceniece R., and Liepinsh E. (2010). The anti-inflammatory and antinociceptive effects of NF-κB inhibitory guanidine derivative ME10092. Int. Immunopharmacol. 10, 455–460 20074673 10.1016/j.intimp.2010.01.006

[30] Otsu N. (1979). A Tlreshold Selection Method from Gray-Level Histograms. IEEE Transactions on Systems, Man, and Cybernetics, 9, 62–66

[31] Hasan I., Dominiak M., Blaszczyszyn A., Bourauel C., Gedrange T., and Heinemann F. (2015). Radiographic evaluation of bone density around immediately loaded implants. Ann. Anat 199, 52–57 24690291 10.1016/j.aanat.2014.02.009

[32] Monje A., Monje F., González-García R., Galindo-Moreno P., Rodriguez-Salvanes F., and Wang H.L. (2014). Comparison between microcomputed tomography and cone-beam computed tomography radiologic bone to assess atrophic posterior maxilla density and microarchitecture. Clin. Oral Implants Res. 25, 723–728 23442126 10.1111/clr.12133

[33] Parsa A., Ibrahim N., Hassan B., van der Stelt P., and Wismeijer D. (2015). Bone quality evaluation at dental implant site using multislice CT, micro-CT, and cone beam CT. Clin. Oral Implants Res. 26, e1–e7 10.1111/clr.12315 24325572

[34] Chan K.H., Mann K.S., Yue C.P., Fan Y.W., and Cheung M. (1990). The significance of skull fracture in acute traumatic intracranial hematomas in adolescents: a prospective study. J. Neurosurg. 72, 189–194 2295916 10.3171/jns.1990.72.2.0189

[35] Servadei F., Ciucci G., Morichetti A., Pagano F., Burzi M., Staffa G., Piazza G., and Taggi F. (1988). Skull fracture as a factor of increased risk in minor head injuries: indication for a broader use of cerebral computed tomography scanning. Surg. Neurol. 30, 364–369 3187881 10.1016/0090-3019(88)90199-1

[36] Lok J., Leung W., Murphy S., Butler W., Noviski N., and Lo E.H. (2011). Intracranial hemorrhage: mechanisms of secondary brain injury, in: *Acta Neurochirurgica*. Supplement. pps. 63–69 10.1007/978-3-7091-0693-8_11 PMC3285293 21725733

[37] Salia S.M., Mersha H.B., Aklilu A.T., Baleh A.S., and Lund-Johansen M. (2018). Predicting dural tear in compound depressed skull fractures: a prospective multicenter correlational study. World Neurosurg. 114, e833–e839 29588245 10.1016/j.wneu.2018.03.095

[38] Braakman R. (1972). Depressed skull fracture: data, treatment, and follow-up in 225 consecutive cases. J. Neurol. Neurosurg. Psychiatry 35, 395–402 5035313 10.1136/jnnp.35.3.395 PMC494082

[39] Agrawal A., Timothy J., Pandit L., and Manju M. (2006). Post-traumatic epilepsy: an overview. Clin. Neurol. Neurosurg. 108, 433–439 16225987 10.1016/j.clineuro.2005.09.001

[40] McColl T.J., Brady R.D., Shultz S.R., Lovick L., Webster K.M., Sun M., McDonald S.J., O'Brien T.J., and Semple B.D. (2018). Mild traumatic brain injury in adolescent mice alters skull bone properties to influence a subsequent brain impact at adulthood: a pilot study. Front. Neurol. 9, 372 29887828 10.3389/fneur.2018.00372 PMC5980957

[41] Lillie E.M., Urban J.E., Lynch S.K., Weaver A.A., and Stitzel J.D. (2016). Evaluation of skull cortical thickness changes with age and sex from computed tomography scans. J. Bone Miner. Res. 31, 299–307 26255873 10.1002/jbmr.2613

[42] Hamel A., Llari M., Piercecchi-Marti M.-D., Adalian P., Leonetti G., and Thollon L. (2013). Effects of fall conditions and biological variability on the mechanism of skull fractures caused by falls. Int. J. Legal Med. 127, 111–118 21984166 10.1007/s00414-011-0627-9

[43] Broglio S.P., Lapointe A., O'Connor K.L., and McCrea M. (2017). Head impact density: a model to explain the elusive concussion threshold. J. Neurotrauma 34, 2675–2683 28381134 10.1089/neu.2016.4767 PMC5647505

[44] Laskowitz D.T., McKenna S.E., Song P., Wang H., Durham L., Yeung N., Christensen D., and Vitek M.P. (2007). COG1410, a novel apolipoprotein E–based peptide, improves functional recovery in a murine model of traumatic brain injury. J. Neurotrauma 24, 1093–1107 17610350 10.1089/neu.2006.0192

[45] Knoblach S.M., Fan L., and Faden A.I. (1999). Early neuronal expression of tumor necrosis factor-α after experimental brain injury contributes to neurological impairment. J. Neuroimmunol. 95, 115–125 10229121 10.1016/s0165-5728(98)00273-2

[46] Woodcock T., and Morganti-Kossmann M.C. (2013). The role of markers of inflammation in traumatic brain injury. Front. Neurol. 4, 18 23459929 10.3389/fneur.2013.00018 PMC3586682

[47] Shohami E., Novikov M., Bass R., Yamin A., and Gallily R. (1994). Closed head injury triggers early production of TNFα and IL-6 by brain tissue. J. Cereb. Blood Flow Metab. 14, 615–619 8014208 10.1038/jcbfm.1994.76

[48] Homsi S., Federico F., Croci N., Palmier B., Plotkine M., Marchand-Leroux C., and Jafarian-Tehrani M. (2009). Minocycline effects on cerebral edema: relations with inflammatory and oxidative stress markers following traumatic brain injury in mice. Brain Res. 1291, 122–132 19631631 10.1016/j.brainres.2009.07.031

[49] Ziebell J.M., Bye N., Semple B.D., Kossmann T., and Morganti-Kossmann M.C. (2011). Attenuated neurological deficit, cell death and lesion volume in Fas-mutant mice is associated with altered neuroinflammation following traumatic brain injury. Brain Res. 1414, 94–105 21871613 10.1016/j.brainres.2011.07.056

[50] Semple B.D., Bye N., Ziebell J.M., and Morganti-Kossmann M.C. (2010). Deficiency of the chemokine receptor CXCR2 attenuates neutrophil infiltration and cortical damage following closed head injury. Neurobiol. Dis. 40, 394–403 20621186 10.1016/j.nbd.2010.06.015

[51] Lagraoui M., Latoche J.R., Cartwright N.G., Sukumar G., Dalgard C.L., and Schaefer B.C. (2012). Controlled cortical impact and craniotomy induce strikingly similar profiles of inflammatory gene expression, but with distinct kinetics. Front. Neurol. 3, 155 23118733 10.3389/fneur.2012.00155 PMC3484408

[52] Cole J.T., Yarnell A., Kean W.S., Gold E., Lewis B., Ren M., McMullen D.C., Jacobowitz D.M., Pollard H.B., O'Neill J.T., Grunberg N.E., Dalgard C.L., Frank J.A., and Watson W.D. (2011). Craniotomy: true sham for traumatic brain injury, or a sham of a sham? J. Neurotrauma 28, 359–369 21190398 10.1089/neu.2010.1427 PMC3057208

[53] Shultz S.R., Sun M., Wright D.K., Brady R.D., Liu S., Beynon S., Schmidt S.F., Kaye A.H., Hamilton J.A., O'Brien T.J., Grills B.L., and Mcdonald S.J. (2015). Tibial fracture exacerbates traumatic brain injury outcomes and neuroinflammation in a novel mouse model of multitrauma. J. Cereb. Blood Flow Metab. 35, 1339–1347 25853909 10.1038/jcbfm.2015.56 PMC4528010

[54] Sun M., Brady R.D., Wright D.K., Kim H.A., Zhang S.R., Sobey C.G., Johnstone MR, O'Brien TJ, Semple BD, McDonald SJ,and Shultz S.R. (2017). Treatment with an interleukin-1 receptor antagonist mitigates neuroinflammation and brain damage after polytrauma. Brain. Behav. Immun. 66, 359–371 28782716 10.1016/j.bbi.2017.08.005

[55] Sun M., Brady R.D., van der Poel C., Apted D., Semple B.D., Church J.E., O'Brien T.J., McDonald S.J., and Shultz S.R. (2018). A concomitant muscle injury does not worsen traumatic brain injury outcomes in mice. Front. Neurol. 9, 1089 30619048 10.3389/fneur.2018.01089 PMC6297867

[56] Lorente L., Martín M.M., Ramos L., Cáceres J.J., Solé-Violán J., Argueso M., Jiménez A., Borreguero-León J.M., Orbe J., Rodríguez J.A., and Páramo J.A. (2015). Serum tissue inhibitor of matrix metalloproteinase-1 levels are associated with mortality in patients with malignant middle cerebral artery infarction. BMC Neurol. 15, 111 26162891 10.1186/s12883-015-0364-7 PMC4499187

[57] Wang L., Kang S., Zou D., Zhan L., Li Z., Zhu W., and Su H. (2016). Bone fracture pre-ischemic stroke exacerbates ischemic cerebral injury in mice. PLoS One 11, e0153835 27089041 10.1371/journal.pone.0153835 PMC4835054

[58] Wang X., Barone F.C., White R.F., and Feuerstein G.Z. (1998). Subtractive cloning identifies tissue inhibitor of matrix metalloproteinase-1 (TIMP-1) increased gene expression following focal stroke. Stroke 29, 516–520 9472898 10.1161/01.str.29.2.516

[59] Rodríguez J.A., Sobrino T., Orbe J., Purroy A., Martínez-Vila E., Castillo J., and Páramo J.A. (2013). proMetalloproteinase-10 is associated with brain damage and clinical outcome in acute ischemic stroke. J. Thromb. Haemost. 11, 1464–1473 23742289 10.1111/jth.12312

[60] Masciantonio M.G., Lee C.K.S., Arpino V., Mehta S., and Gill S.E. (2017). The balance between metalloproteinases and TIMPs, in: *Progress in Molecular Biology and Translational Science*. pps. 101–131 10.1016/bs.pmbts.2017.01.001 28413026

[61] White T.E., Ford G.D., Surles-Zeigler M.C., Gates A.S., LaPlaca M.C., and Ford B.D. (2013). Gene expression patterns following unilateral traumatic brain injury reveals a local pro-inflammatory and remote anti-inflammatory response. BMC Genomics 14, 282 23617241 10.1186/1471-2164-14-282 PMC3669032

[62] Gardner J. and Ghorpade A. (2003). Tissue inhibitor of metalloproteinase (TIMP)-1: the TIMPed balance of matrix metalloproteinases in the central nervous system. J. Neurosci. Res. 74, 801–806 14648584 10.1002/jnr.10835 PMC3857704

