# Supplementary material for: Skull Fractures Induce Neuroinflammation and Worsen Outcomes after Closed Head Injury in Mice
Source: J Neurotrauma. 2019 Dec 20;37(2):295–304. doi: 10.1089/neu.2019.6524 (PMC6964812; doi:10.1089/neu.2019.6524)
Supplement: Supplemental data [file Supp_Table1.pdf]

Supplementary Data

Supplementary Table S1. F Values, Degrees of Freedom and p Values for Gene Expression Data Analysis (Ordinary Two-Way Analyis of Variance)

Brain structure		*TIMP-1*	*TNF-α*	*IL-6*	*IL-1β*	*MMP-9*
Ipsilateral hippocampus	Time effect	*F(3, 60) = 5.8, p < 0.01	*F(3, 62) = 6.7, p < 0.01	*F(3, 61) = 8.6, p < 0.0001	*F(3, 61) = 3.5, p < 0.05	F(3, 62) = 1.7, p > 0.05
	Group effect	*F(2, 60) = 17.7, p < 0.0001	*F(2, 62) = 6.2, p < 0.01	F(2, 61) = 0.2, p > 0.05	F(2, 61) = 0.2, p > 0.05	F(2, 62) = 2.3, p > 0.05
	Time × group interaction	*F(6, 60) = 2.6, p < 0.05	*F(6, 62) = 3.4, p < 0.01	F(6, 61) = 2.0, p > 0.05	F(6, 61) = 0.4, p > 0.05	F(6, 62) = 1.7, p < 0.05
Contralateral hippocampus	Time effect	F(3, 64) = 2.5, p > 0.05	*F(3, 65) = 5.0, p < 0.01	F(3, 65) = 1.7, p > 0.05	*F(3, 65) = 4.9, p < 0.01	*F(3, 63) = 3.6, p < 0.05
	Group effect	*F(2, 64) = 6.9, p < 0.05	F(2, 65) = 0.6, p > 0.05	*F(2, 65) = 4.9, p < 0.05	F(2, 65) = 1.0, p > 0.05	F(2, 63) = 0.4, p > 0.05
	Time × group interaction	F(6, 64) = 1.1, p > 0.05	F(6, 65) = 0.3, p > 0.05	F(6, 65) = 0.6, p > 0.05	F(6, 65) = 0.2, p > 0.05	F(6, 63) = 0.04, p > 0.05
Ipsilateral striatum	Time effect	*F(3, 61) = 3.7, p < 0.05	*F(3, 57) = 4.0, p < 0.05	F(3, 60) = 1.9, P > 0.05	*F(3, 56) = 4.3, p < 0.01	*F(3, 61) = 5.6, p < 0.01
	Group effect	*F(2, 61) = 12.7, p < 0.0001	*F(2, 57) = 5.0, p < 0.01	F(2, 60) = 0.3, p > 0.05	*F(2, 56) = 3.3, p < 0.05	F(2, 61) = 0.6, p > 0.05
	Time × group interaction	F(6, 61) = 1.1, p > 0.05	F(6, 57) = 0.6, p > 0.05	F(6, 60) = 0.9, p > 0.05	F(6, 56) = 1.0, p > 0.05	F(6, 61) = 0.1, p > 0.05
Contralateral striatum	Time effect	*F(3, 56) = 5.0, p < 0.01	*F(3, 55) = 4.0, p < 0.05	F(3, 57) = 1.0, p > 0.05	F(3, 53) = 1.6, p > 0.05	F(3, 54) = 1.4, p > 0.05
	Group effect	*F(2, 56) = 10.3, p < 0.05	*F(2, 55) = 8.9, p < 0.01	F(2, 57) = 1.0, p > 0.05	*F(2, 53) = 4.7, p < 0.05	F(2, 54) = 2.2, p > 0.05
	Time × group interaction	*F(6, 56) = 2.4, p < 0.05	F(6, 55) = 2.1, p > 0.05	F(6, 57) = 0.7, p > 0.05	F(6, 53) = 1.0, p > 0.05	F(6, 54) = 0.3, p > 0.05

TIMP-1, tissue inhibitor of metalloproteinase; TNF-α, tumor necrosis factor-α; IL, interleukin; MMP-9, matrixmetalloproteinase-9.
*Significant effect.
